# A massive abdominal wall desmoid tumor occurring in a laparotomy scar: A case report

**DOI:** 10.1186/1477-7819-9-35

**Published:** 2011-03-22

**Authors:** Joseph K Wanjeri, Collins JO Opeya

**Affiliations:** 1 Department of Surgery, School of Medicine, University of Nairobi, Kenya

## Abstract

**Introduction:**

Desmoid tumors are benign but locally aggressive tumors of mesenchymal origin which are poorly circumscribed, infiltrate the surrounding tissue, lack a true capsule and are composed of abundant collagen. History of trauma to the site of tumor origin is elicited in up to 1 in 4 cases and they most commonly develop in the anterior abdominal wall and shoulder girdle but they can arise in any skeletal muscle. The clinical behavior and natural history of desmoid tumors are unpredictable and management is difficult with many issues remaining controversial, mainly regarding early detection, the role, type and timing of surgery and the value of non-operative therapies.

**Case presentation:**

We report a case of a 23 year old male referred from a district hospital to a national referral hospital in Kenya, after developing a huge abdominal wall desmoid tumor following laparotomy for a blunt abdominal injury fourteen months earlier. The tumor was successfully excised and the abdominal wall defect reconstructed using a vicryl/prolene mesh and a unilateral groin flap. The patient had a non-eventful recovery and was discharged through radiotherapy clinic.

**Conclusion:**

Wide margin tumor excision alone is a reasonable option in the management of desmoid tumors.

## Introduction

Desmoid tumors account for 0.3% of all neoplasms and less than 3% of all soft tissue tumors with the estimated incidence in the general population being 2-4 per million of population per year [1-3]. Affected patients mostly fall within the age range 10-40 years with those younger than 10 years or older than 44 being affected rarely [1].

The myofibroblast is the cell considered responsible for the development of desmoid tumors but the mechanisms of development and regulation of their growth are unknown [1,4,5]. Trauma may have a triggering effect in the development of the tumors and the tumors may be solitary or multiple[1,6]. Extra-abdominal and intra-abdominal forms of the disease have been distinguished and in abdominal wall disease, the tumor is usually confined to the musculature and the overlying aponeurosis or fascia but the neoplasm may infiltrate the surrounding tissue up to 2-3 cm outside the palpable tumor [3,7,8].

The clinical behaviour and natural history of desmoid tumors remain unpredictable and enigmatic: while in some patients it progresses rapidly and aggressively, in others it is more indolent and may remain stable without any subsequent problem for sometime [6]. Most desmoid tumors are slow-growing neoplasms that do not metastasize but aggressively invade surrounding tissues and organs or may compress surrounding structures [3,6]. Desmoid tumors often arise from the rectus abdominis muscle in postpartum women and in scars of previous abdominal incisions [9,10]. Imaging methods including ultrasonography, Computed Tomography (CT) and Magnetic Resonance Imaging (MRI) are used for diagnosis and evaluation of these tumors [9].

## Case presentation

The patient was a 23 year old male who presented with an anterior abdominal wall mass in December 2007 following an emergency laparotomy for blunt abdominal trauma in June 2006. He was referred to Kenyatta National Hospital from a district hospital where an incisional biopsy had been done and reported as benign fibromatosis. There was no family history of Familial Adenomatous Polyposis (FAP), colorectal disease or similar condition in any of the close relatives.

**Figure 1:**
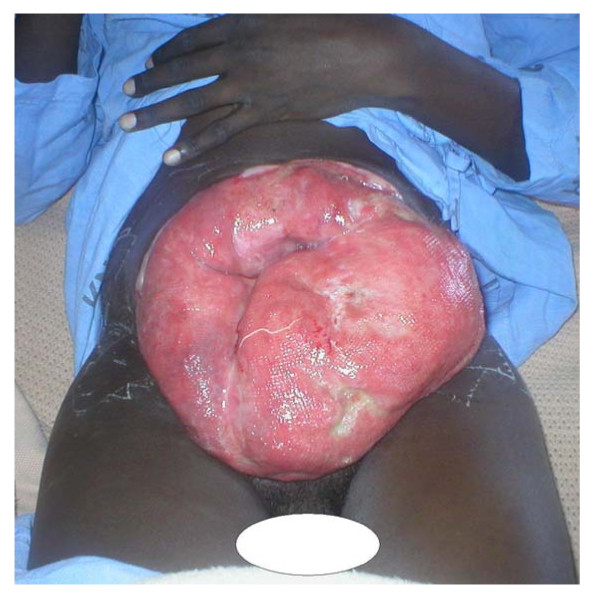
**The ulcerated anterior abdominal wall tumor with everted edges**.

**Figure 2:**
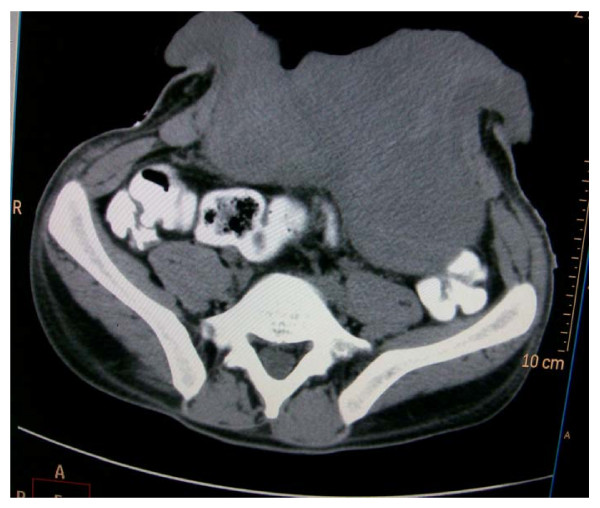
**CT scan of the abdomen showing the anterior abdominal wall tumor**.

On examination, his general condition was fair and he had a huge ulcerated anterior abdominal wall mass with everted edges measuring 16 cm × 20 cm (figure 1) which was reported as desmoid tumor after incisional biopsy was done. An abdominal CT scan showed hepatomegaly and a mass measuring 16 cm × 15 cm × 4.6 cm confined to the anterior abdominal wall with an intra-abdominal extension but no involvement of intra abdominal organs (figure 2). Neither genetic testing for the Adenomatous Polyposis Coli (APC) gene mutation nor screening with colonoscopy for adenomatous polyposis coli or colorectal cancer was performed.

**Figure 3:**
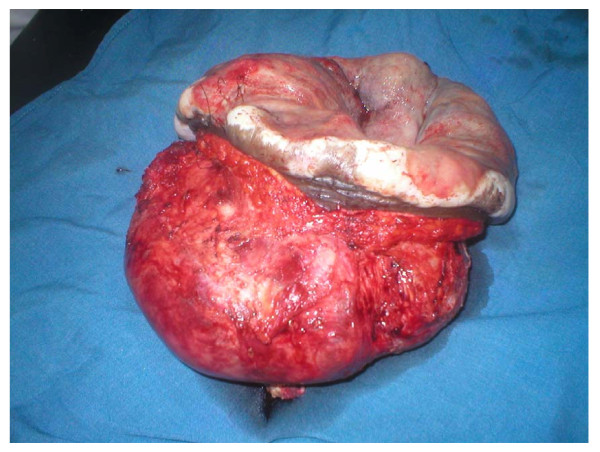
**The excised anterior abdominal wall tumor**.

**Figure 4:**
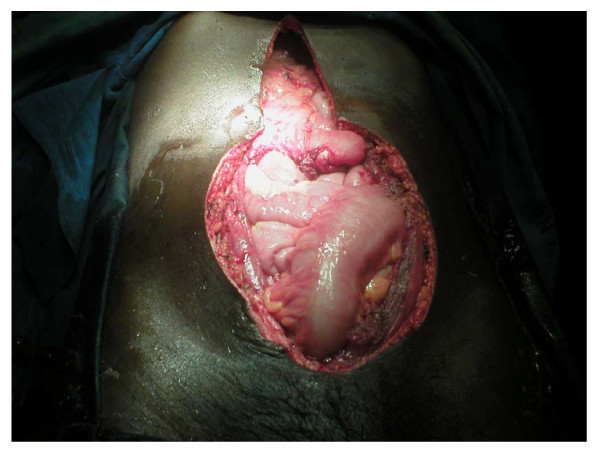
**The resulting anterior abdominal wall defect following the excision of the tumor**.

**Figure 5:**
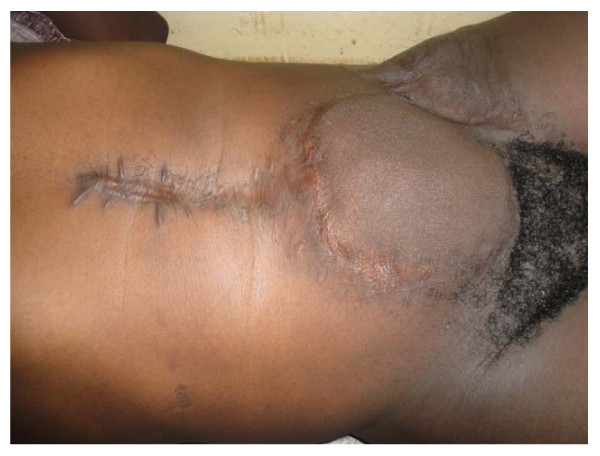
**The anterior abdominal wall 30 months after excision of the tumor. Note the incisional hernia**.

In January 2008, the tumor weighing an estimated two kilograms (figure 3) was excised with a 3 cm macroscopic margin and the resultant defect (figure 4) reconstructed with a vicryl/prolene mesh. A left local fasciocutaneous groin flap was rotated to cover the mesh and the secondary defect on the left groin area covered with a split thickness skin graft. The patient had a non-eventful postoperative recovery period and was discharged through radiotherapy clinic but he missed his appointment. The authors traced him thirty months later and found him without any sign of recurrence but he had an incisional hernia at the site of tumor excision and repair (figure 5).

## Discussion

Desmoid tumors are monoclonal fibroblastic proliferations arising in musculoaponeurotic structures. They are benign but aggressive tumors of mesenchymal origin, forming a heterogenous group of pathologic entities resulting from the proliferation of well-differentiated fibroblasts [11,12]. At microscopy, desmoid tumors are poorly circumscribed, infiltrate the surrounding tissue, lack a true capsule and are composed of abundant collagen surrounding poorly circumscribed bundles of elongated, slender, spindle-shaped cells of uniform appearance [9].

Most desmoid tumors occur sporadically but about 2-5% commonly occur in the abdominal cavity or abdominal wall in association with FAP [6]. Inheritance (or new mutation) of one copy of APC tumor suppressor gene is the cause of FAP and the two commonest causes of deaths in these patients are duodenal cancer and desmoid tumors [6]. In FAP associated cases, desmoid tumors represent an extra-colonic manifestation of polyposis syndrome [13]. Every patient with desmoid tumor should therefore be evaluated for the presence of associated polyposis syndrome by taking a detailed family history, performing colonoscopy and possibly upper GastroIntestinal (GI) endoscopy [1]. The patient in this report did not have a family history of polyposis and colonoscopy was not done due to financial constraints.

Management of patients with desmoid tumors is difficult and many issues remain controversial, mainly regarding early detection, the role, type and timing of surgery, and the value of non-operative therapies [1]. The main difficulty in treatment is due to the fact that these tumors are histologically benign but have a high propensity for local recurrence [3]. Women have been found to be more likely to require multiple desmoid tumor resections than men, an observation which supports the hypothesis that estrogens stimulate desmoid growth [6]. Estrogen's regulatory role is supported further by the higher incidence of desmoid tumors in women during their reproductive years, the apparent tendency of tumors to develop during pregnancy or soon after, their occasional disappearance after menopause, the proliferation of similar lesions in laboratory animals by estrogen administration and the potential benefit of anti-estrogen drugs [1,3,13,14].

There are no good randomized clinical trials of treatment for desmoid tumors and most studies are based on small case series. The effects of treatment are further compounded by the variable natural history of the disease with some tumors apparently regressing or remaining stable even without treatment [1].

Management of desmoid tumors involves a multidisciplinary approach with rapidly growing tumors being managed more aggressively [3]. Our patient was managed by a team of general surgeons, a plastic surgeon, a radio-oncologist, a psychological counselor, a social worker, nurses and a pathologist. Surgery is the mainstay of treatment in the management of extra-abdominal desmoid tumors and resection of abdominal wall tumors especially can be performed safely [6,9]. Radical (free margin) excision as in this case report offers the best chance for cure and of avoiding local recurrence [3]. Unfortunately, radical surgery is not always a straightforward procedure because of the tumor's extent and invasiveness. Superficial abdominal wall desmoid tumors should be resected before they become large in order to avoid having large soft tissue defects with resultant complicated and technically more demanding reconstruction [15-17]. Abdominal wall reconstruction can be achieved by direct repair (with sutures), and by using synthetic materials (meshes) or myocutaneous flaps when the defect is large as in this case report [16,18,19]. Surgery may also be required for the management of complications such as hemorrhage, bowel perforation, hollow visceral obstruction, peritonitis or sepsis.

Radiation therapy has been used mainly for the treatment of extra-abdominal desmoid tumors and has resulted in improvement of local control of desmoid tumors by reducing local recurrence rates [18-21]. External-beam irradiation or brachytherapy has been used alone in patients with inoperable lesions, but it has been associated with high failure rates [11,19]. Radiotherapy may also be used either before surgery or as adjuvant therapy following incomplete (non-radical) surgical resection [2,3,6,22]. The patient in this case report was referred to radiotherapy unit and was scheduled to receive radiotherapy following the presumed complete excision of the tumor but he defaulted treatment. He was traced by the authors many months post-operatively and clinical examination revealed an incisional hernia (figure 5) but no evidence of local tumor recurrence at the excision site indicating that wide excision alone may be adequate in the management of desmoid tumors.

The role of radiofrequency ablation in the management of these tumors is still under investigation and could be considered in selected patients and only when other treatment modalities have failed [21]. Percutaneous chemical ablation with acetic acid under radiological guidance is another therapeutic option and unproven treatments with pirfenidone, interferon alpha and glivec (imatinib, 800 mg/d) may be effective, but only anecdotal reports or small series have been published so far [23-27]. Gene transfer therapy is also a field of intensive research currently in the management of desmoid tumors [28].

## Conclusion

Desmoid tumors are rare in clinical practice and their management remains quite challenging due to their variable clinical behavior. Wide excision with tumor free margins may be adequate in the management of abdominal wall tumors as shown by the current case report.

## Competing interests

The authors declare that they have no competing interests.

## Authors' contributions

CJOO drafted most of the initial manuscript and traced the patient in November 2010, JKW drafted parts of the manuscript and critically revised the whole manuscript before submission to the editor and publisher. Both authors read and approved the final manuscript.

## Consent

Written informed consent was obtained from the patient for publication of this report and accompanying images. A copy of the written consent is available for review by the Editor-in-Chief of this journal.
